# Effect of a fixed combination of ripasudil and brimonidine on aqueous humor dynamics in mice

**DOI:** 10.1038/s41598-024-58212-6

**Published:** 2024-04-03

**Authors:** Reiko Yamagishi-Kimura, Megumi Honjo, Makoto Aihara

**Affiliations:** Department of Ophthalmology, Graduate School of Medicine, The University of Tokyo, 7-3-1 Hongo, Bunkyo-ku, Tokyo, 1138655 Japan

**Keywords:** Physiology, Medical research

## Abstract

Ripasudil–brimonidine fixed-dose combination (K-232) simultaneously targets three different intraocular pressure (IOP) lowering mechanisms, increasing trabecular meshwork outflow and uveoscleral outflow, and reducing aqueous humor production Vascularly, ripasudil induces transient vasodilation, brimonidine transient vasoconstriction. Investigating effects on IOP, aqueous dynamics, and EVP in mice eyes by microneedle and constant-pressure perfusion methods, and on cytoskeletal and fibrotic proteins changes in HTM cells by a gel contraction assay and immunocytochemistry. Ripasudil, K-232, and brimonidine droplets significantly reduced IOP at 30 min, with K-232 sustaining the effect at 60 min. For EVP, only K-232 exhibited reduced EVP until 60 min after instillation. In vitro, ripasudil inhibited gel contractility and TGFβ2-induced fibrotic changes, whereas brimonidine did not. K-232 significantly lowered IOPs in mice by combining the effects of ripasudil and brimonidine. Brimonidine alone also showed IOP reductions with enhanced outflow facility, and the drug did not interfere with the effects of ripasudil on the trabecular meshwork outflow; K-232 and ripasudil alone both significantly lowered the EVP and enhanced outflow facility, demonstrating that K-232 efficiently reduces IOPs.

## Introduction

Lowering intraocular pressure (IOP) is a reliable way to treat glaucoma^1,2^, and drug therapy with eye drops is considered the first choice for open-angle glaucoma^3,4^. The IOP is maintained within a normal range through a balance between the production and outflow of aqueous humor. This liquid is produced by non-pigmented, ciliary epithelial cells^5,6^. it circulates in the anterior chamber and then drains through two routes: the trabecular meshwork (or conventional) and the uveoscleral (or unconventional) outflow pathways^5,7^ Under physiological conditions, the trabecular meshwork is the major outflow pathway, accounting for up to 90% of aqueous outflow in aged human eyes, according to the results of direct measurements using a tracer^8^. Through this route, the aqueous humor drains from the anterior chamber sequentially through the uveal and corneoscleral trabecular meshwork beams, the juxtacanalicular connective tissue (JCT), and Schlemm’s canal (SC). After entering the SC lumen, the aqueous humor drains into the collector channels, intrascleral plexus, episcleral veins, and finally into the blood circulation. Experimental evidence suggests that most of the trabecular outflow resistance is generated in the JCT region and inner walls of the SC^9,10^. However, some studies have suggested that one-third to one-half of the total outflow resistance is distal to the SC^11,12^. Episcleral vein pressure (EVP), which contributes to distal outflow resistance and has been deemed relevant in patients with normal-tension glaucoma (NTG), primary open-angle glaucoma (POAG)^13,14^, or secondary glaucoma^13,15^, The EVP is believed to play a crucial role in the regulation of the IOP^16^.

Ripasudil hydrochloride hydrate (ripasudil 0.4%), a Rho-associated coiled coil-containing protein kinase (ROCK) inhibitor, is an IOP-lowering medication. It functions by increasing aqueous humor outflow via the trabecular outflow pathway, modulating the trabecular meshwork cell cytoskeleton, adjusting the extracellular matrix composition, and increasing the SC permeability^17^. The pharmacologic actions of ROCK inhibitors include modulating the motility, contractility, adhesion, and shape of most local cell types, resulting in smooth muscle relaxation and morphological changes in corneal endothelial cells, in addition to IOP reduction^18–20^. Netarsudil, a ROCK inhibitor available in the United States and other countries, causes dilation of episcleral veins^21^.

Brimonidine is an agonist of α2-adrenergic receptors (α2 agonist) that reduces the IOP by suppressing aqueous humor production and promoting uveoscleral aqueous outflow^22–24^. One mechanism of action of α2 agonists is vasoconstriction, resulting in the reduction of aqueous inflow and transient but apparent bleaching of ocular surface vessels.

A fixed-dose combination of ripasudil (0.4%) and brimonidine tartrate (0.1%), K-232, has been developed in Japan to enhance patient adherence to treatment^25,26^. The fixed-dose combination drugs have various benefit for sustainable patient’s adherence in point of the number of drops and bottles, the dose of preservatives, adequate interval acquisition between different kind of drugs, or economics.

Ripasudil–brimonidine fixed-dose combination (; RBFC) has theoretically ideal IOP-reducing properties because it targets three dynamics factors: enhancement of both trabecular and uveoscleral outflow and suppression of aqueous humor production. However, the contrasting vascular reactions from ROCK inhibition and α2-adrenergic stimulation might antagonize their own IOP-reduction effects.

In this study, we investigated the interaction in the efficacy of RBFC (development code K-232) in terms of IOP lowering, impact on the EVP and aqueous humor outflow as a treatment for glaucoma using a mouse model. We also compared the histopathological effects of the relevant drugs on cultured human trabecular meshwork (HTM) cells in vitro.

## Results

### Effect of K-232 ophthalmic solution, ripasudil, and brimonidine on IOP

To elucidate the IOP-lowering effect of K-232, we used a microneedle to measure the IOPs of mice at different time points after adding eye drops of commercially available presentations containing ripasudil (Rip), brimonidine (Bri), mixtures of both drugs (Rip/Bri), or a fixed combination of ripasudil and brimonidine(K-232) (n = 5–7) or saline (control).

**Figure 1:**
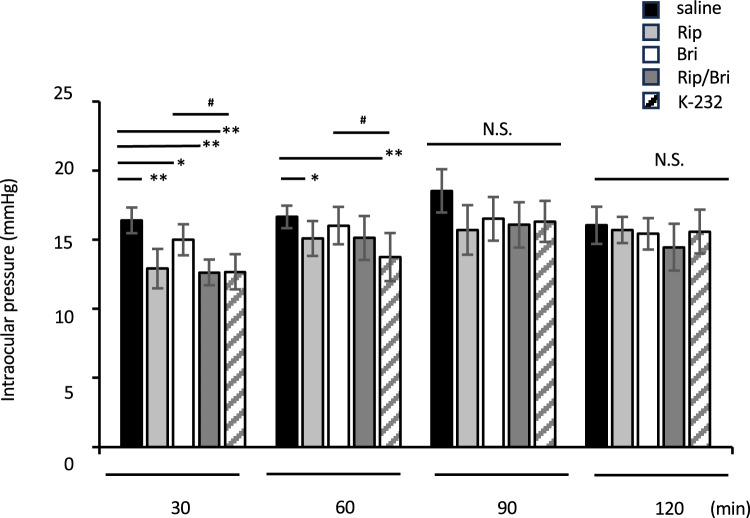
Time courses of IOP reductions induced by either 0.4% ripasudil, 0.1% (w/v) brimonidine, or K-232 in WT mice. Data are expressed as means ± SDs (n = 5–7). *, **p < 0.05 or 0.01, respectively, for each drug treatment vs. saline treatment comparison. #*p* < 0.05 for each drug treatment vs. brimonidine treatment (Tukey test).

As shown in Fig. 1, the mean IOPs 30 min after adding saline, Rip, Bri, Rip/Bri, and K-232 were 16.4 ± 0.93, 12.9 ± 1.42, 14.4 ± 0.53, 12.6 ± 0.93, and 12.7 ± 1.27 mmHg, respectively. See the figure for the values at the other time points. In summary, the IOPs after treatment with any product were significantly lower than that of controls at 30 min. In addition, there were significant differences between Bri and Rip/Bri. At 60 min, there were significant differences between controls and Rip and K-232 and also between K-232 and Bri. There were no significant differences between groups at the 90 or 120 min time points, despite a tendency toward lower IOPs for all products at 90 min.

### Outflow facility measurements

**Figure 2:**
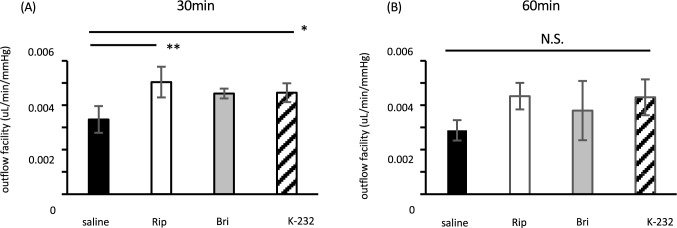
Outflow facility after treatment of mice with either 0.4% ripasudil, 0.1% brimonidine, or K-232. Outflow facilities were measured at the time of IOP reduction after instillation. Data are expressed as means ± SDs (n = 4–5). *, **p < 0.05 or 0.01, respectively, for each drug treatment vs. saline treatment comparison (Tukey test). (**A**) 30 min after instillation. (**B**) 60 min after instillation.

To elucidate the IOP-lowering mechanisms, we measured outflow facilities at 30 and 60 min after drug treatment, corresponding to times when we had observed significant differences between controls and the K-232 group. The values are shown in Fig. 2. There were significant differences between controls and both Rip and K-232 at 30 min; at 60 min, there was still evidence of enhanced outflow in both treatment groups but without statistical significance.

### EVP measurements

**Figure 3:**
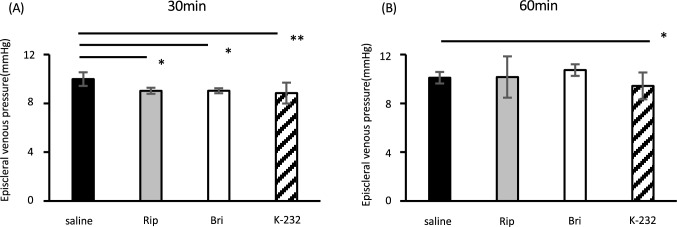
EVP after treatment with either 0.4% ripasudil, 0.1% brimonidine, or K-232. EVPs were measured at the time of IOP reduction after instillation. Data are expressed as means ± SDs (n = 5–7). *, **p < 0.05 or 0.01, respectively, for drug-treated versus saline-treated eyes (Tukey test). (**A**) 30 min after instillation. (**B**) 60 min after instillation.

After observing significant differences in the IOP-lowering and outflow-enhancing effects of drugs 30 and 60 min after instillation, we decided to measure EVPs. The values are given in Fig. 3. There were significant differences between all products and controls at 30 min, but only between K-232 and controls at 60 min.

### HTM-mediated collagen gel contraction assays

**Figure 4:**
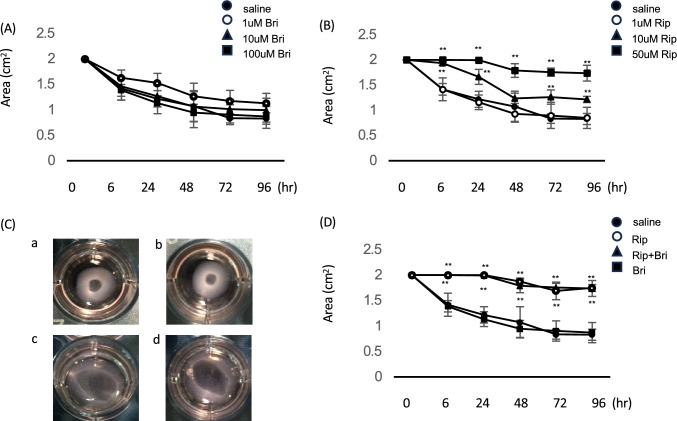
Effects of ripasudil and brimonidine on HTM-mediated collagen gel contractility. Collagen gels were incubated with ripasudil, brimonidine, or ripasudil/brimonidine for 96 h. Data are expressed as means ± SDs (n = 4). **p < 0.01 for drug-treated versus saline-treated eyes (Tukey test). (**A**) 1, 10, and 100 µM brimonidine treatment. (**B**) 1, 10, and 50 µM ripasudil treatment. (**C**) Representative images of the gels; a, saline; b, brimonidine; c, ripasudil; and d, ripasudil/brimonidine. (**D**) Concomitant effect of ripasudil and brimonidine on HTM-mediated collagen gel contractility.

We performed collagen gel contraction assays to evaluate the effect of each drug on HTM-mediated gel contraction. We found similar contraction values for controls and brimonidine-treated cells at all tested concentrations. By contrast, 10 and 50 µM Rip significantly inhibited TGFβ2-induced HTM cells contractions after 24, 48, 72 and 96 h. Similarly, TGFβ2-induced contractions were significantly suppressed after Bri/Rip (100 µM/10 µM) treatments at each time point (Fig. 4).

### Effects on TGFβ2-induced cytoskeletal and fibrotic changes in HTM cells

**Figure 5:**
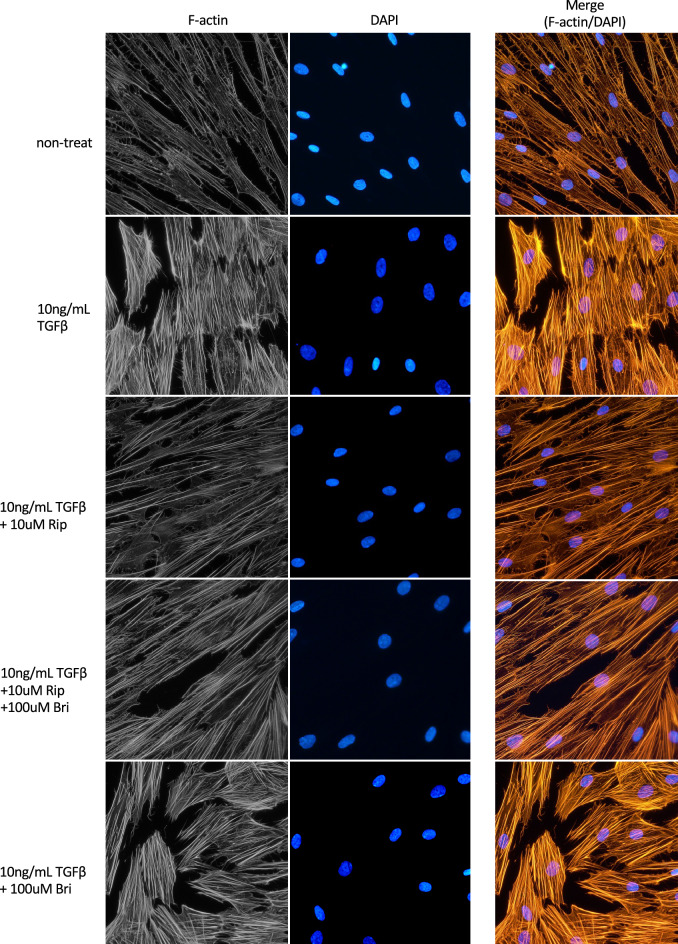
Effects of ripasudil and brimonidine on cytoskeletal changes in HTM cells. The effects of ripasudil and brimonidine on TGFβ2-induced cytoskeletal changes in HTM cells at 24 h were evaluated via immunocytochemistry. The left panels show cells stained for F-actin. The middle panels show cell nuclei counterstained with 4’,6-diamidino-2-phenylindole (DAPI; blue). The right panels show merged images. TGFβ2 significantly induced cytoskeletal changes in HTM cells and these changes were suppressed by treatment with either ripasudil or ripasudil/brimonidine.

**Figure 6:**
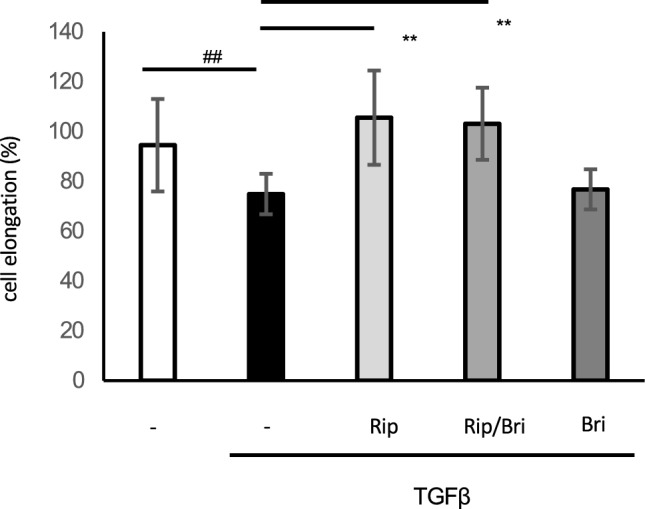
Effects of ripasudil and brimonidine on HTM cells in terms of cell elongation. The effects of ripasudil and brimonidine on TGFβ2-induced cell elongation in F-actin-stained cells at 24 h were evaluated using IN Cell Analyzer. Data are expressed as means ± SDs (n = 12–17). ##*p* < 0.01 for nontreated cells versus TGFβ2-treated cells, **p < 0.01 for TGFβ2- + ripasudil- or ripasudil/brimonidine-treated cells versus TGFβ2-treated cells (Tukey test).

**Figure 7:**
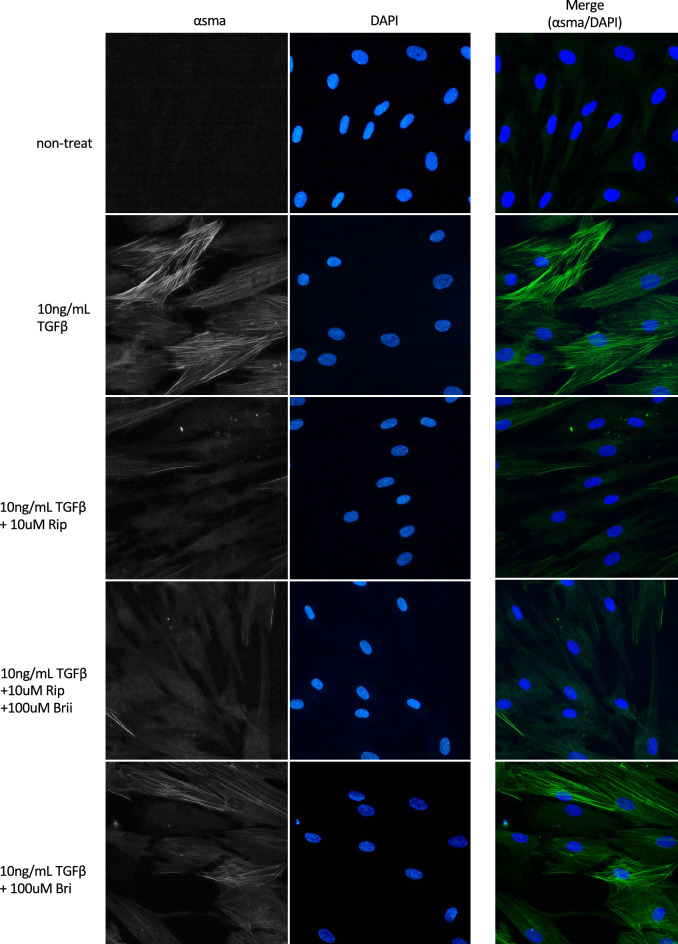
Effects of ripasudil and brimonidine on fibrotic changes in HTM cells. The effect of ripasudil and brimonidine on the TGFβ2-induced fibrotic changes observed in HTM cells after 24 h of treatment were evaluated via immunocytochemistry. The left panels show cells stained for αSMA. The middle panels show cell nuclei counterstained with 4’,6-diamidino-2-phenylindole (DAPI; blue). The right panels show merged images. TGFβ2 significantly induced fibrotic changes in HTM cells and these changes were suppressed by treatment with ripasudil and ripasudil/brimonidine.

**Figure 8:**
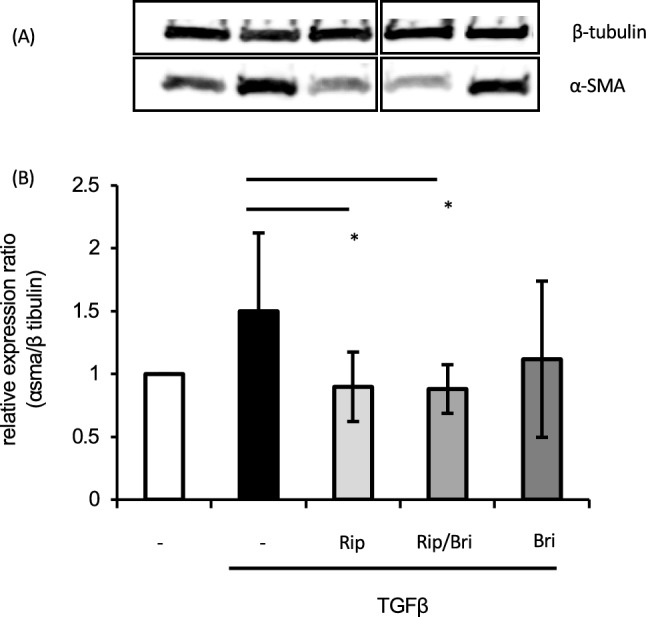
Effects of ripasudil and brimonidine on TGFβ2-induced αSMA expression in HTM cells. HTM cells were treated with 10 ng/mL TGFβ2 and either ripasudil, brimonidine, or ripasudil/brimonidine, or TGFβ2 alone for 24 h. Effects of ripasudil and brimonidine on the expression of αSMA. (**A**) Representative Western blots of β-tubulin and αSMA. Data are presented as means ± SDs, n = 5 *p < 0.05, TGFβ2- + ripasudil- or ripasudil/brimonidine-treated versus TGFβ2 alone (Tukey test). Original blots are presented in Supplementary Fig. S2.

We investigated cytoskeletal and fibrotic changes in HTM cells via immunocytochemistry. TGFβ2 treatment increased the expression of fibrotic proteins, such as α-smooth muscle actin (αSMA), and induced cytoskeletal changes characterized by actin bundles with F-actin staining. The TGFβ2-induced cytoskeletal changes were mitigated by treatment with 10 nM Rip. However, treatment with 100 µM Bri did not affect the TGFβ2-induced changes. We found similar levels of suppression of cytoskeletal changes to those obtained with ripasudil after Rip/Bri treatments (Fig. 5). In addition, we assessed cell elongation by staining F-actin and analyzing images IN Cell Analyzer 2200 (GE Healthcare). We found that TGFβ2 treatment significantly reduced cell elongation compared to controls. Moreover, the cell elongation reduction by TGFβ2 was significantly suppressed by ripasudil or Rip/Bri treatment but not Bri alone (Fig. 6). By contrast, according to immunostaining experiments, TGFβ2 treatment increased the expression of the fibrotic protein αSMA, but Rip or Rip/Bri treatments inhibited this increase (Fig. 7). Moreover, our Western blot results confirmed that the TGFβ2-induced αSMA expression was suppressed by Rip or Rip/Bri treatments (Fig. 8).

## Discussion

We evaluated the IOP-lowering efficacy of a ripasudil–brimonidine fixed-dose combination, and elucidated its IOP-lowering mechanisms. We confirmed that the fixed combination effectively reduces IOPs. In addition, there was significant EVP reduction as well as enhanced outflow facility after K-232 and ripasudil treatments. To the best of our knowledge, this is the first report of an estimated evaluation of EVP measurement in the mouse eye; our results are in accordance with the established mechanisms of ROCK inhibitors. Brimonidine did not alter the effects of ripasudil on the trabecular meshwork outflow pathway when applied simultaneously (both in vivo and in vitro).

As shown in Fig. 1, all tested drugs significantly reduced the IOP 30 min after administration, while ripasudil and K-232 significantly maintained the reduction at 60 min. These results suggest that ROCK inhibitors provide longer sustained IOP-lowering effects than brimonidine after a single dose. Therefore, the fixed dose combination may also offer a long effect.

We evaluated outflow facility and showed that K-232 increased outflow facility significantly after instillation as well as ripasudil did (Fig. 2). This was expected because ROCK inhibitor reduces IOP by enhancing outflow facility^17^. However, 60 min after K-232 and ripasudil administration, when we observed a significant reduction in IOP, the increase in outflow facility was not statistically significant, despite showing an upward trend. This relatively short response, which aligns well with published reports, can be attributed to the small size and thin sclera of mouse eyes, along with physiological differences compared to human eyes. If there are no species differences in the metabolism or binding rate of the drug used and normal mice are used in the experiment, it is known that the turnover rate was about two times higher in mice than in humans^27–29^ or IOP reduction responses in mice were fairly quickly^30,31^.

We also found that all products reduced EVPs in the mouse eye (Fig. 3). As far as we know, these data are the first to show that Rho inhibition alone leads to reduced EVPs in mouse eyes. Netarsudil (AR13324), a ROCK inhibitor with norepinephrine transport inhibitory activity, has been reported to lower EVPs in rabbits^32^, and it induces dilation of episcleral vessels in enucleated human eyes, but these effects have been suggested to be mediated mostly by the norepinephrine transport inhibitory activity that causes vasoconstriction^32,33^. The precise mechanisms regulating episcleral circulation remain unclear^34,35^ because the interplay between the blood flow and vascular resistance in the episcleral supply arterioles, various arteriovenous anastomoses (AVAs), and muscular veins^36–41^ is complex. Pharmacologically induced vasodilation and vasoconstriction of the episcleral vasculature can alter the blood flow through AVAs, affecting the EVP, the flow of aqueous into the episcleral veins, and the IOP^40^. Overall vasodilation has been suggested to engorge the AVAs, fill the venules with arterial blood, and increase the EVP, resulting in outflow resistance and IOP elevation^40,42^. By contrast, vasoconstriction has been suggested to reduce the venous perfusion volume and decrease the episcleral venous blood flow, two actions that would enhance the flow of aqueous humor in the veins.

ROCK inhibitors act on the vascular smooth muscle generating distinct vasorelaxant effects: conjunctival hyperemia is often induced by topical ripasudil instillation, peaks at approximately 5–15 min after administration, and generally resolves within 2 h^43^. Although the general vasodilation of the episcleral vasculature (which includes the AVAs) has been suggested to cause increases in both the EVP and IOP, as mentioned above, AVA closure, which reduces blood flow from the arterioles to the venules, may reduce the EVP and IOP^21,40,42^. We hypothesized that a ROCK inhibitor would produce selective vasodilation of the episcleral veins only, without opening the AVAs, and that ROCK inhibitor-induced vasodilation of the episcleral vasculature might reduce the resistance to aqueous outflow and lower the IOP. A study in which the dilation of the sclero-conjunctival vasculature was evaluated using anterior segment-optical coherence tomography angiography (AS-OCTA) supports this hypothesis. In that study, vasodilation associated with ripasudil treatment was primarily caused by selective vasodilation of deep episcleral veins^44^. In addition, the changes in vessel density detected by AS-OCTA were significantly associated with IOP reductions. Moreover, in other studies, topical ripasudil treatment for 1–3 months resulted in dilation of vessels in the scleral vascular plexus^45^, suggesting a possible increase in aqueous-venous drain into the episcleral plexus. In a clinical study, the use of ripasudil in patients with OAG after circumferential incision of the Schlemm’s canal by a 360° suture trabeculotomy ab interno produced significant additional IOP reductions, supporting the idea that ripasudil produces distal outflow^46^. The mechanisms of the episcleral vasculature and EVP effects of ROCK inhibitors resulting in IOP reductions remain unclear. Further studies are needed to clarify the association between episcleral vasculature dilation and EVP/IOP.

Brimonidine is a known vasoconstrictor, and it has been shown to decrease the EVP in rabbits^47^; our results in mice support that finding. In addition to the EVP reduction 30 min after drug administration, it resulted in a slightly increased trend of enhanced outflow facility (Figs. 2 and 3). Its IOP-reduction mechanisms during long-term treatments are due to the suppression of aqueous humor production or enhancement of uveoscleral outflow; fluorophotometry and tomography studies in human eyes have reported that brimonidine does not affect the aqueous flow, outflow facility, or EVP^23,24^. Thus, different results have been obtained in rabbits and humans with respect to blood flow. According to previous reports, α2 receptors have been classified into subtypes A–C^48,49^. These subtypes have been shown to have different effects on blood flow, with subtype 2A decreasing blood flow and subtype 2B increasing blood flow^50^. Subtypes B and C have been shown to be predominant in humans and all three are present in the rabbit^51^, but there are no reports on mice. Taken together, we speculate that the differences in the effects of brimonidine on ciliary blood flow in humans, rabbits, and mice may be related to the differences in the distribution of α2 receptor subtypes in the ciliary vasculature.

In addition, regarding the effects of brimonidine on aqueous outflow and IOP, an acute effect on aqueous dynamics has been reported in human eyes after a single topical drop. The effect is due to brimonidine stimulating α2-receptors within blood vessels of the uvea or ciliary body, causing vasoconstriction, decreases in ciliary blood flow, and decreases in aqueous production^52^. However, it is also reported that even though vasoconstriction may persist overtime, the aqueous flow would be attenuated and return toward its previous value due to the fact that receptors responsible for vasoconstriction may be highly sensitive to pharmacologic stimulation acutely but may lose some of this sensitivity during long-term continued treatment^53^. The suggested mechanisms for the EVP reductions we observed after ripasudil or brimonidine treatments are controversial; therefore, further investigation is needed for clarification. Notably, K-232 treatment led to more sustained EVP reductions than ripasudil or brimonidine treatment alone. In addition, in experiments using HTM cells, we confirmed that brimonidine did not interfere with the trabecular effects of ripasudil. Thus, we speculate that neither brimonidine nor ripasudil hinders the trabecular aqueous outflow or EVP reduction mechanisms of each other.

Our study had some limitations. First, we measured IOPs, outflow facility, and EVPs using a mouse model but did not further investigate aqueous humor production or uveoscleral outflow. Mouse eyes are so small that measuring aqueous humor production or evaluating its detailed outflow is difficult. Future studies should examine the details of aqueous humor production and other outflow pathways. Second, the mechanisms by which ripasudil and K-232 lower EVPs remain unknown. In addition, the results of the present study suggested that brimonidine also may enhanced outflow facility. Although there is a concern that the outflow pathway after TM may be different from normal due to the slightly higher intraocular pressure used in our two-level constant pressure method, there have been several previous studies using similar methods^54–56^ and studies examining the correlation between IOP values and outflow facility measurements have shown that linearity is maintained within the range of IOP values used in this study^57^. So far, several methods have been reported for the measurement of outflow facility in the past, but there are several factors that can affect the results of each method^58,59^ and no uniform method has been established. Therefore, in the present study, we investigated the outflow facility using the method stated, but improving the accuracy of measurement of outflow facility (especially measurement using mice) remains an important issue to be resolved in the future. Third, the mechanisms by which ripasudil and K-232 lower EVPs remain unknown. This is a subject for a future study. Finally, we studied the effects of K-232 on the IOP, outflow facility, and EVP in normal mice using single drops of drug but did not study the effects of K-232 on actual patients with glaucoma. We plan to investigate this in the future. Finally, in this study, in vivo experiments tended not to show significant differences in the later time point. We suspected that individual variability might be a major cause of this, and it could be attributable to the low power of the experiment itself. In particular, we could not follow time-dependent changes in the same individual because of our experimental methods, and it might have made it difficult all the more to detect significant differences. Another reason could be the characteristics of brimonidine as a drug affecting the contralateral eye. Normally, we use the non-treated eye as a control to minimize the influence of individual differences on the test results. However, in the case of brimonidine, the contralateral eye could not be used as a control, therefore the individual variability might have affected on the test results.

In conclusion, K-232 effectively reduced both the IOP and EVP, while enhancing outflow facility, in the eyes of mice. Thus, this combination shows promise as a valuable treatment for glaucoma.

## Materials and methods

### Materials

For in vivo mouse studies, we used the GLA-ALPHA^®^ combination ophthalmic solution (the fixed-dose combination of 0.4% ripasudil and 0.1% brimonidine, K-232 provided by KOWA Company Ltd.; Aichi, Japan) and the ophthalmic solutions GLANATEC^®^ (0.4% ripasudil; Rip) and AIPHAGAN^®^ (0.1% brimonidine; Bri) purchased commercially. For in vitro studies, ripasudil was provided by KOWA Company Ltd. (Aichi, Japan) and brimonidine was purchased from FUJIFILM Wako Pure Chemical Corporation (Osaka, Japan).

### Animals

All animals were treated in accordance with the ARVO Statement for the Use of Animals in Ophthalmic and Vision Research and the dictates of our local Animal Use Committee at the University of Tokyo. The protocol for animal experiments used in this study was approved by the University of Tokyo's Animal Ethics Committee. All experiments were performed by relevant named guidelines, regulations, and the ARRIVE guidelines. Male C57BL/6 J (wild type, WT) and ddY (wild type, WT) mice were purchased from Japan SLC (Shizuoka, Japan). The housing temperature was maintained at 21 °C with a 12 h light/dark cycle starting at 6:00 AM. After purchase, all mice had access to food and water ad libitum in a conventional animal room in our laboratory for at least 1 week before the scheduled experimental date. For all experiments, we used 8- to 10-week-old male mice (body weight range, 18–24 g).

### Preparation and adding ophthalmic solution

Rip, Bri and K-232 ophthalmic solutions were stored at room temperature. We also prepared a mixture of 0.4% ripasudil and 0.1% brimonidine (Rip/Bri) to use in in vivo studies. Using a micropipette, we applied 3 μL of each eye drop solution or saline (control) topically (in a masked manner) to one eye selected at random. For experiments requiring concomitant use of both drugs, we delivered specific eyedrops at 5 min intervals.

### Mouse IOP measurements

IOPs were measured using microneedles^29^. Briefly, the mice were anesthetized using an intraperitoneal injection of a mixture of ketamine (100 mg/kg, Ketalar; DAIICHI SANKYO COMPANY, Tokyo, Japan) and xylazine (9 mg/kg; Seractal, Bayer, Berlin, Germany) prepared at room temperature. We administered anesthesia using a 29-gauge needle. A timer was started immediately after the injection, and we measured IOPs within 4–5 min after administration of anesthesia in all animals. The borosilicate glass microneedle (75–100 μm tip diameter and 1.0 mm outer diameter, 25° bevel angle) was connected to a pressure transducer and the data were sent to a data acquisition and analysis system. We measured IOPs in both eyes of each anesthetized mouse, placing a microneedle in the anterior chamber of the eye.

### Outflow facility measurements

To clarify the mechanism by which K-232 reduces IOPs, we measured the outflow facility 30 and 60 min after adding either a drug or saline. We determined the time of measurement as the time at which we found significant IOP reduction. To measure outflow facility, we used a two-level constant-pressure perfusion method expressed as a C value^29,60^. Briefly, an infusion needle identical to the one used for IOP measurements was inserted into the anterior chamber and connected to a reservoir filled with artificial aqueous humor via a pressure transducer. The liquid surface height in the chamber was maintained at 25 or 35 mmHg for a steady-state period of 10 min (H_25_ or H_35_, respectively). The inner cross-sectional area of the reservoir (S) was calculated based on the inner diameter. The volumes of artificial aqueous humor infused per min at 25 or 35 mmHg were denoted as V_25_ and V_35_. The total outflow facility (C_total,_ μL/min/mm Hg) was calculated based on the following formula: C_total_ = (V_35_ − V_25_)/10 = [S(H_35_ − H_25_)]/100. The C_total_ was the sum of C_conv_ and C_uveo_, where C_conv_ is the outflow facility of the pressure-dependent system and C_uveo_ is the uveoscleral system (assumed to be less pressure-dependent).

### Episcleral venous pressure measurements in mice

To clarify the mechanism by which K-232 reduces IOPs, we measured EVPs 30 and 60 min after injecting the eyes with either drug or saline. To measure the superior scleral vein pressure, we followed a modified version of a published protocol^29,61^ Briefly, we used ddY mice for EVP measurement and a glass needle connected to a transducer and a reservoir bag. After inserting the needle into the anterior chamber of the eye of an anesthetized mouse, we raised or lowered the reservoir bag connected to the transducer to artificially control the pressure in the anterior chamber. Initially, we adjusted the height of the reservoir bag to match the IOP of the mouse, and then we progressively lowered it at a rate of 0.5 mmHg/min while observing the vascular pattern of the surface of the mouse eye under a microscope at 60× magnification. We defined the EVP as the pressure at which venous blood flowed back into Schlemm’s canal and recorded its value.

### Preparation of human trabecular meshwork cells, and drug treatments

Primary HTM cells were isolated from human donor eyes (donor No. 21–0025 100 (50y, Female), 21–0023 200 (36y, Male) and 21–0023 100 (36y, Male)) obtained from the Rocky Mountain Lions Eye Bank (Colorado, U.S.) and characterized. (supplemental Fig. S1)^62^ Only well-characterized normal HTM cells, in which Dexamethasone (Dex)-induced myocilin (MYOC) upregulation was confirmed with quantitative qRT-PCR from passages 3 through 5 were used in our studies (Supplementary Figure. S1A). Furthermore, for the HTM cell characterization, Dex-induced MYOC upregulation and immunocytochemistry using antibodies against Aquaporin 1 (AQP-1), Collagen Type IV (COL4A1), Matrix Gla Protein (MGP), tissue inhibitor of metalloproteinase (TIMP)-3, vimentin, and desmin was also performed according to previous reports (Supplementary Fig. S1B)^63,64^. All donor tissues were obtained and managed in line with the guidelines of the Declaration of Helsinki for research involving human tissues. Cells were cultured in Dulbecco’s modified Eagle’s medium (DMEM) containing 10% fetal bovine serum and antibiotic–antimycotic solution (100×) (Sigma-Aldrich, St. Louis, MO, USA) at 37 °C and 5% CO2. We used only well-characterized normal HTM cells from passages 3–6 in subsequent experiments.

The ripasudil concentrations in aqueous humor 30–60 min after a single adding ripasudil ophthalmic solution (1.0%, w/v) have been reported to be approximately 10 µg/mL (~ 25 µM) in rabbits and 1 µg/mL (~ 2.5 µM) in monkeys^65^. Another study showed that brimonidine levels in aqueous humor 2 h after brimonidine instillation (0.2%, w/v) were 0.647 ± 0.062 µg/mL (~ 1.4 µM) in rabbits^66^ and 336.0 ± 276.2 nM in humans^67^. While we are aware of potential interspecies differences in aqueous humor concentrations, we deliberately chose ripasudil concentrations higher than 1 µM and a brimonidine concentration higher than 1 µM for our in vitro experiments with HTM cells, unless otherwise specified. We set a 10 ng/mL concentration of the stimulant TGFβ2 to obtain a positive baseline control for cytoskeletal and fibrotic changes in HTM cells.

### Collagen gel contraction assay, immunostaining, and Western blotting using HTM cells

#### Collagen gel contraction assays

We used a Cell Contraction Assay kit (Nitta Gelatin, Osaka, Japan) as described to assess collagen gel contractility by HTM cells^68^. Briefly, we generated a collagen gel matrix in 24-well plates as instructed by the manufacturer. HTM cells were cultured on the collagen gel until confluent. The tops of the collagen gel were stimulated, and the gels were detached from the walls of the culture wells. We photographed the gel areas at 0, 6, 24, 48, 72, and 96 h and analyzed them using ImageJ software (National Institutes of Health, Bethesda, MD, USA). We masked the data inputs for the analysis. Changes in the areas are given as contraction percentages compared to start of the incubation period. We used TGFβ2 stimulation to obtain a positive control for gel contraction and observed its inhibition using each drug alone or drug combinations.

#### Immunostaining

Immunocytochemistry was performed as described in a previous study^68^. We used the primary antibodies anti-alpha-smooth muscle actin (α-SMA, 1:400; Dako, Agilent, Santa Clara, CA, USA) and rhodamine phalloidin (7:1000; Thermo Fisher Scientific, Waltham, MA, USA). Alexa Fluor 488- and Alexa Fluor 594-conjugated secondary antibodies (1:1000) were purchased from Thermo Fisher Scientific. Images were obtained using a BX51 fluorescence microscope (Olympus, Tokyo, Japan).

#### Western blotting

Western blotting was performed as published^69^. Briefly, we collected cell lysates in RIPA buffer (Thermo Fisher Scientific) containing protease inhibitors (Roche Diagnostics, Basel, Switzerland) after treatment. Protein concentrations were determined using a BCA Protein Assay kit (Thermo Fisher Scientific) according to the manufacturer’s protocol with bovine serum albumin as a standard. Protein extracts were separated by SDS-PAGE and transferred to PVDF membranes (Bio-Rad Laboratories). Then we immersed the membranes in a solution containing primary antibodies overnight at 4 °C. The primary antibodies were anti-alpha smooth muscle actin (1:1000; DAKO) and anti-β-tubulin (1:1000; Wako Pure Chemical Industries, Osaka, Japan). After washing the membranes, we incubated them in a solution with horseradish peroxidase-conjugated anti-mouse or anti-rabbit secondary antibody (1:2000–1:5000; Thermo Fisher Scientific) for 1 h at room temperature. The proteins in the membranes were made to react with ECL substrate (Thermo Fisher Scientific), and we placed the membranes in an ImageQuant LAS 4000 mini-imager (GE Healthcare, Chicago, IL, USA) to visualize the bands and quantify them using ImageJ software (ver. 1.49, NIH, Bethesda, MD, USA).

#### Data analysis and statistics

All results are presented as means ± standard deviations (SDs). Statistical analyses were performed using JMP Pro version 11 software. All data are the means of at least three independent experiments. We deemed differences as statistically significant at *P*-values < 0.05, as determined by ANOVA, Student t-test, Tukey, or Dunnett test results.

### Supplementary Information

Supplementary Figures.

## Data Availability

All data generated or analysed during the current study available from the corresponding author on reasonable request.

## References

[1] Heijl A (2002). Reduction of intraocular pressure and glaucoma progression: Results from the early manifest glaucoma trial. Arch. Ophthalmol..

[2] Collaborative Normal-Tension Glaucoma Study Group (1998). Comparison of glaucomatous progression between untreated patients with normal-tension glaucoma and patients with therapeutically reduced intraocular pressures. Am. J. Ophthalmol..

[3] Wang T, Cao L, Jiang Q, Zhang T (2021). Topical medication therapy for glaucoma and ocular hypertension. Front. Pharmacol..

[4] Lee DA, Higginbotham EJ (2005). Glaucoma and its treatment: A review. Am. J. Health Syst. Pharm..

[5] Goel M, Picciani RG, Lee RK, Bhattacharya SK (2010). Aqueous humor dynamics: A review. Open Ophthalmol. J..

[6] Aptel F, Weinreb RN, Chiquet C, Mansouri K (2016). 24-h monitoring devices and nyctohemeral rhythms of intraocular pressure. Prog. Retin. Eye Res..

[7] Križaj, D. What is glaucoma? In: Webvision: The Organization of the Retina and Visual System [Internet]. (eds. Kolb, H., Fernandez, E., Nelson, R.) (University of Utah Health Sciences Center; 1995). 21413389

[8] Bill A, Phillips CI (1971). Uveoscleral drainage of aqueous humour in human eyes. Exp. Eye Res..

[9] Grant WM (1963). Experimental aqueous perfusion in enucleated human eyes. Arch. Ophthalmol..

[10] Mäepea O, Bill A (1989). The pressures in the episcleral veins, Schlemm’s canal and the trabecular meshwork in monkeys: Effects of changes in intraocular pressure. Exp. Eye Res..

[11] Schuman JS, Chang W, Wang N, de Kater AW, Allingham RR (1999). Excimer laser effects on outflow facility and outflow pathway morphology. Invest. Ophthalmol. Vis. Sci..

[12] Rosenquist R, Epstein D, Melamed S, Johnson M, Grant WM (1989). Outflow resistance of enucleated human eyes at two different perfusion pressures and different extents of trabeculotomy. Curr. Eye Res..

[13] Selbach JM, Posielek K, Steuhl KP, Kremmer S (2005). Episcleral venous pressure in untreated primary open-angle and normal-tension glaucoma. Ophthalmologica.

[14] Talusan ED, Schwartz B (1981). Episcleral venous pressure. Differences between normal, ocular hypertensive, and primary open angle glaucomas. Arch. Ophthalmol..

[15] Jørgensen JS, Guthoff R (1988). The role of episcleral venous pressure in the development of secondary glaucomas. Klin. Monbl. Augenheilkd..

[16] Machiele R, Motlagh M, Patel BC (2023). StatPearls.

[17] Honjo M, Tanihara H (2018). Impact of the clinical use of ROCK inhibitor on the pathogenesis and treatment of glaucoma. Jpn. J. Ophthalmol..

[18] Yamagishi-Kimura R (2018). Interaction between pilocarpine and ripasudil on intraocular pressure, pupil diameter, and the aqueous-outflow pathway. Invest. Ophthalmol. Vis. Sci..

[19] Kaneko Y (2016). Effects of K-115 (Ripasudil), a novel ROCK inhibitor, on trabecular meshwork and Schlemm’s canal endothelial cells. Sci. Rep..

[20] Nakagawa H, Koizumi N, Okumura N, Suganami H, Kinoshita S (2015). Morphological changes of human corneal endothelial cells after rho-associated kinase inhibitor eye drop (Ripasudil) administration: A prospective open-label clinical study. PLoS One.

[21] Ren R (2016). Netarsudil increases outflow facility in human eyes through multiple mechanisms. Invest. Ophthalmol. Vis. Sci..

[22] Burke J, Schwartz M (1996). Preclinical evaluation of brimonidine. Surv. Ophthalmol..

[23] Fan S, Agrawal A, Gulati V, Neely DG, Toris CB (2014). Daytime and nighttime effects of brimonidine on IOP and aqueous humor dynamics in participants with ocular hypertension. J. Glaucoma.

[24] Toris CB, Gleason ML, Camras CB, Yablonski ME (1995). Effects of brimonidine on aqueous humor dynamics in human eyes. Arch. Ophthalmol..

[25] Tanihara H (2023). Crossover randomized study of pharmacologic effects of Ripasudil–Brimonidine fixed-dose combination versus ripasudil or brimonidine. Adv. Ther..

[26] Tanihara H (2023). Ripasudil–Brimonidine fixed-dose combination versus ripasudil or brimonidine: Two phase 3 randomized clinical trials. Am. J. Ophthalmol..

[27] Kazemi A (2019). Effect of timolol on aqueous humor outflow facility in healthy human eyes. Am. J. Ophthalmol..

[28] Toris CB, Yablonski ME, Wang YL, Camras CB (1999). Aqueous humor dynamics in the aging human eye. Am. J. Ophthalmol..

[29] Aihara M, Lindsey JD, Weinreb RN (2003). Aqueous humor dynamics in mice. Invest. Ophthalmol. Vis. Sci..

[30] Larsson LI (2001). Intraocular pressure over 24 h after single-dose administration of latanoprost 0.005% in healthy volunteers. A randomized, double-masked, placebo controlled, cross-over single center study. Acta Ophthalmol. Scand..

[31] Aihara M, Lindsey JD, Weinreb RN (2002). Reduction of intraocular pressure in mouse eyes treated with latanoprost. Invest. Ophthalmol. Vis. Sci..

[32] Kiel JW, Kopczynski CC (2015). Effect of AR-13324 on episcleral venous pressure in Dutch belted rabbits. J. Ocul. Pharmacol. Ther..

[33] Bönisch H, Brüss M (2006). The norepinephrine transporter in physiology and disease. Handb. Exp. Pharmacol..

[34] Ascher KW (2018). The aqueous veins: I. Physiologic importance of the visible elimination of intraocular fluid. Am. J. Ophthalmol..

[35] Ascher K.W. Aqueous veins: II. Local pharmacologic effects on aqueous veins III. Glaucoma and aqueous veins. *Am. J. Opthalmol.* **25**, 1301–1315 (1942).

[36] Selbach JM, Schönfelder U, Funk RH (1998). Arteriovenous anastomoses of the episcleral vasculature in the rabbit and rat eye. J. Glaucoma.

[37] Rohen JW, Funk RH (1994). Functional morphology of the episcleral vasculature in rabbits and dogs: Presence of arteriovenous anastomoses. J. Glaucoma.

[38] Funk RH, Rohen JW (1994). In vivo observations of the episcleral vasculature in the albino rabbit. J. Glaucoma.

[39] Funk RH, Rohen JW (1996). Scanning electron microscopic study of episcleral arteriovenous anastomoses in the owl and cynomolgus monkey. Curr. Eye Res..

[40] Funk RH, Gehr J, Rohen JW (1996). Short-term hemodynamic changes in episcleral arteriovenous anastomoses correlate with venous pressure and IOP changes in the albino rabbit. Curr. Eye Res..

[41] Rohen JW, Funk RH (1994). Vasculature of the anterior eye segment. Prog. Retin. Eye Res..

[42] Lee SS, Robinson MR, Weinreb RN (2019). Episcleral venous pressure and the ocular hypotensive effects of topical and intracameral prostaglandin analogs. J. Glaucoma.

[43] Terao E (2017). Time course of conjunctival hyperemia induced by a rho-kinase inhibitor anti-glaucoma eye drop: Ripasudil 0.4. Curr. Eye Res..

[44] Akagi T (2020). Short-term effects of different types of anti-glaucoma eyedrop on the sclero-conjunctival vasculature assessed using anterior segment OCTA in normal human eyes: A pilot study. J. Clin. Med..

[45] Limratchatamorn B, Asakawa K, Mashimo K, Uga S, Ishikawa H (2019). Effects of 0.4% ripasudil hydrochloride hydrate on morphological changes in rabbit eyes. Int. J. Ophthalmol..

[46] Sato T, Kawaji T (2021). Effects of ripasudil on open-angle glaucoma after circumferential suture trabeculotomy Ab interno. J. Clin. Med..

[47] Reitsamer HA, Posey M, Kiel JW (2006). Effects of a topical alpha2 adrenergic agonist on ciliary blood flow and aqueous production in rabbits. Exp. Eye Res..

[48] Civantos Calzada B, de Aleixandre Artiñano A (2001). Alpha-adrenoceptor subtypes. Pharmacol. Res..

[49] Alberts P (1993). Subtype classification of presynaptic alpha 2-adrenoceptors. Gen. Pharmacol..

[50] Philipp M, Brede M, Hein L (2002). Physiological significance of alpha(2)-adrenergic receptor subtype diversity: One receptor is not enough. Am. J. Physiol. Regul. Integr. Comp. Physiol..

[51] Huang Y, Gil DW, Vanscheeuwijck P, Stamer WD, Regan JW (1995). Localization of alpha 2-adrenergic receptor subtypes in the anterior segment of the human eye with selective antibodies. Invest. Ophthalmol. Vis. Sci..

[52] Toris CB, Camras CB, Yablonski ME (1999). Acute versus chronic effects of brimonidine on aqueous humor dynamics in ocular hypertensive patients. Am. J. Ophthalmol..

[53] Van Buskirk EM, Bacon DR, Fahrenbach WH (1990). Ciliary vasoconstriction after topical adrenergic drugs. Am. J. Ophthalmol..

[54] Melena J, Zalduegui A, Arcocha P, Santafé J, Segarra J (1999). Topical verapamil lowers outflow facility in the rabbit eye. J. Ocul. Pharmacol. Ther..

[55] Tokushige H (2007). Effects of topical administration of y-39983, a selective rho-associated protein kinase inhibitor, on ocular tissues in rabbits and monkeys. Invest. Ophthalmol. Vis. Sci..

[56] Dai Y (2009). Outflow facility in mice with a targeted type I collagen mutation. Invest. Ophthalmol. Vis. Sci..

[57] Yelenskiy A (2017). Total outflow facility in live C57BL/6 mice of different age. Biomed. Hub..

[58] Boussommier-Calleja A (2015). Physical factors affecting outflow facility measurements in mice. Invest. Ophthalmol. Vis. Sci..

[59] Tian B, Hu Y, Gabelt BT, Kaufman PL (2006). Factors affecting outflow facility calculations. Exp. Eye Res..

[60] Crowston JG, Aihara M, Lindsey JD, Weinreb RN (2004). Effect of latanoprost on outflow facility in the mouse. Invest. Ophthalmol. Vis. Sci.

[61] Aihara M, Lindsey JD, Weinreb RN (2003). Episcleral venous pressure of mouse eye and effect of body position. Curr. Eye Res..

[62] Keller KE (2018). Consensus recommendations for trabecular meshwork cell isolation, characterization and culture. Exp. Eye Res..

[63] Stamer WD, Clark AF (2017). The many faces of the trabecular meshwork cell. Exp. Eye Res..

[64] Zhu W, Godwin CR, Cheng L, Scheetz TE, Kuehn MH (2020). Transplantation of iPSC-TM stimulates division of trabecular meshwork cells in human eyes. Sci. Rep..

[65] Isobe T, Kasai T, Kawai H (2016). Ocular penetration and pharmacokinetics of ripasudil following topical administration to rabbits. J. Ocul. Pharmacol. Ther..

[66] Dong JQ (2004). Effects of the preservative purite on the bioavailability of brimonidine in the aqueous humor of rabbits. J. Ocul. Pharmacol. Ther..

[67] Takamura Y (2015). Vitreous and aqueous concentrations of brimonidine following topical application of brimonidine tartrate 0.1% ophthalmic solution in humans. J. Ocul. Pharmacol. Ther..

[68] Honjo M (2007). Potential role of Rho-associated protein kinase inhibitor Y-27632 in glaucoma filtration surgery. Invest. Ophthalmol. Vis. Sci..

[69] Honjo M (2001). Effects of protein kinase inhibitor, HA1077, on intraocular pressure and outflow facility in rabbit eyes. Arch. Ophthalmol..

