# Supplementary material for: Effect of a fixed combination of ripasudil and brimonidine on aqueous humor dynamics in mice
Source: Sci Rep. 2024 Apr 3;14:7861. doi: 10.1038/s41598-024-58212-6 (PMC10991514; doi:10.1038/s41598-024-58212-6)

**Effect of a fixed combination of Ripasudil and Brimonidine on aqueous humor dynamics in mice.**

Reiko Yamagishi-Kimura^1^ PhD, *Megumi Honjo^1^ MD PhD, Makoto Aihara^1^ MD PhD

^1^Department of Ophthalmology, The University of Tokyo School of Medicine,

Japan.

*Corresponding author:

Megumi Honjo, M.D., Ph.D.

Address:

Department of Ophthalmology,

The University of Tokyo School of Medicine,

7-3-1 Hongo Bunkyo-ku, Tokyo, 113-8655, Japan

Telephone number: +81-3-3815-5411

Fax number: +81-3-3817-0798

honjomegumi@gmail.com

Supplementary Figure S1

Only well-characterized normal HTM cells, in which Dexamethasone (Dex)-induced myocilin (MYOC) upregulation was confirmed with quantitative qRT-PCR from passages 3 through 5 were used in our studies (Supplementary Figure. S1(A)). Furthermore, for the HTM cell characterization, Dex-induced MYOC upregulation and immunocytochemistry using antibodies against Aquaporin 1 (AQP-1), Collagen Type IV (COL4A1), Matrix Gla Protein (MGP), tissue inhibitor of metalloproteinase (TIMP)-3, vimentin, and desmin was also performed according to previous reports (Supplementary Figure S1(B)).


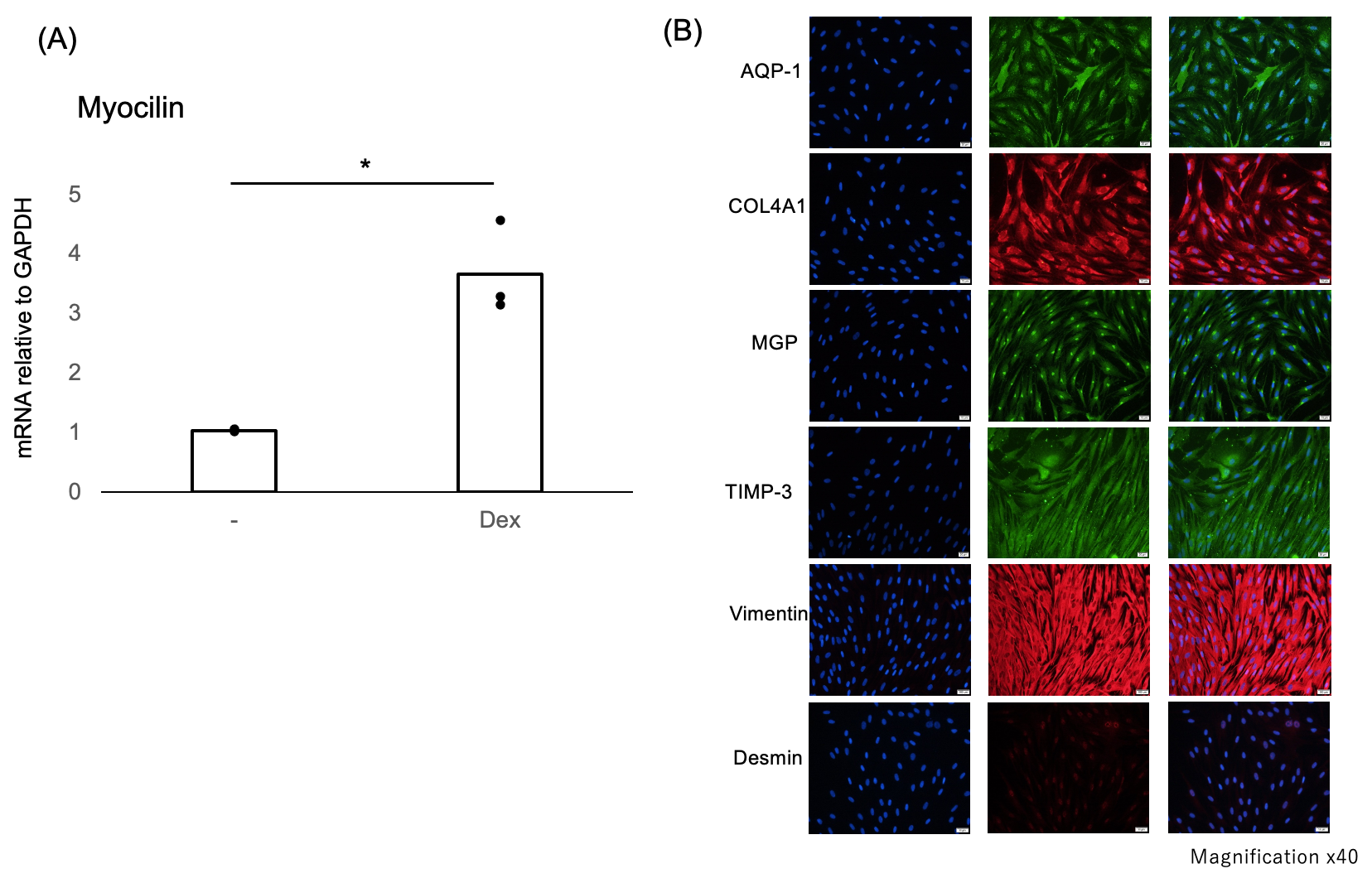


Supplementary Figure S2

Effects of ripasudil and brimonidine on TGFβ2-induced αSMA expression in HTM cells. HTM cells were treated with 10 ng/mL TGFβ2 and either ripasudil, brimonidine, or ripasudil/brimonidine, or TGFβ2 alone for 24 h. Western blot gel images of the effects of ripasudil and brimonidine on the expression of αSMA (A) and β-tubulin (B). The X and Y bands are for drug-treatments other than those used in this study. The membrane was cut up and down at 25 kDa. prior to hybridization with antibodies.


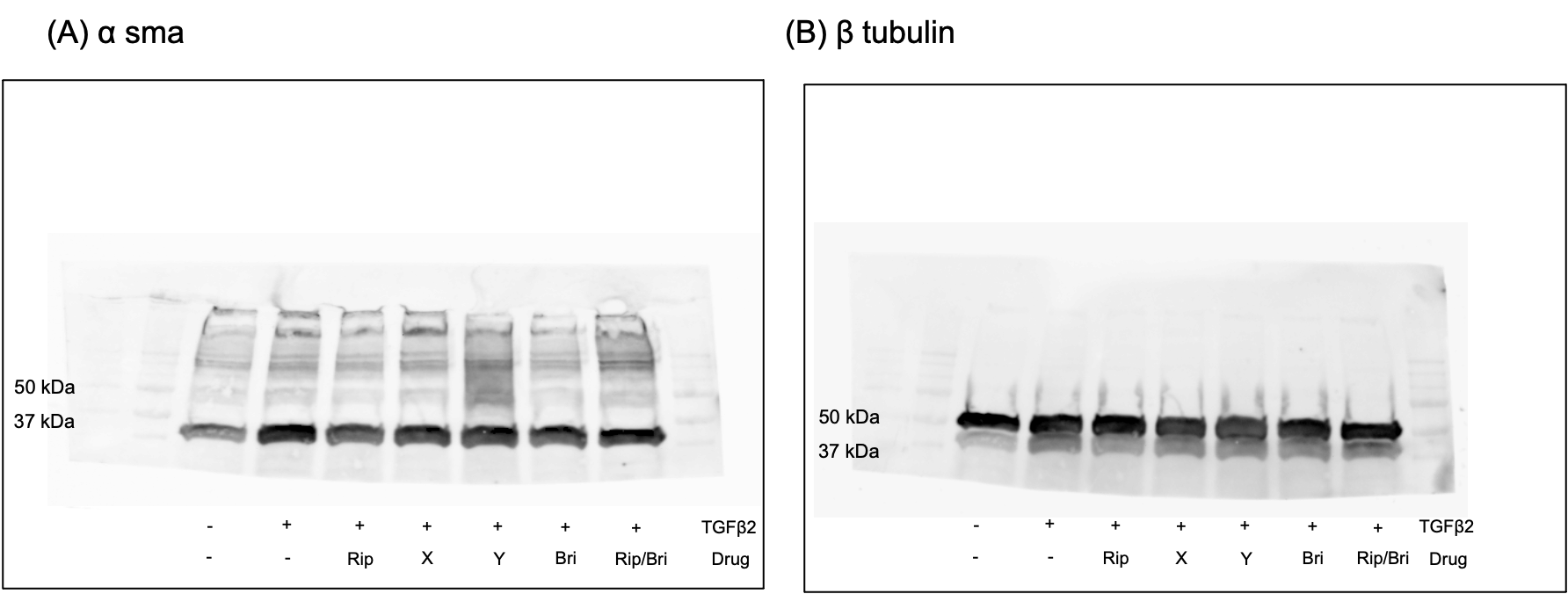

Supplement: Supplementary file 1 — Supplementary Figures. [file 41598_2024_58212_MOESM1_ESM.docx]
